# Supplementary material for: Potential Antitumor Effect of α-Mangostin against Rat Mammary Gland Tumors Induced by LA7 Cells
Source: Int J Mol Sci. 2023 Jun 17;24(12):10283. doi: 10.3390/ijms241210283 (PMC10299034; doi:10.3390/ijms241210283)
Supplement: Supplementary file 1 [file ijms-24-10283-s001.zip › ijms-2328863-supplementary.pdf]

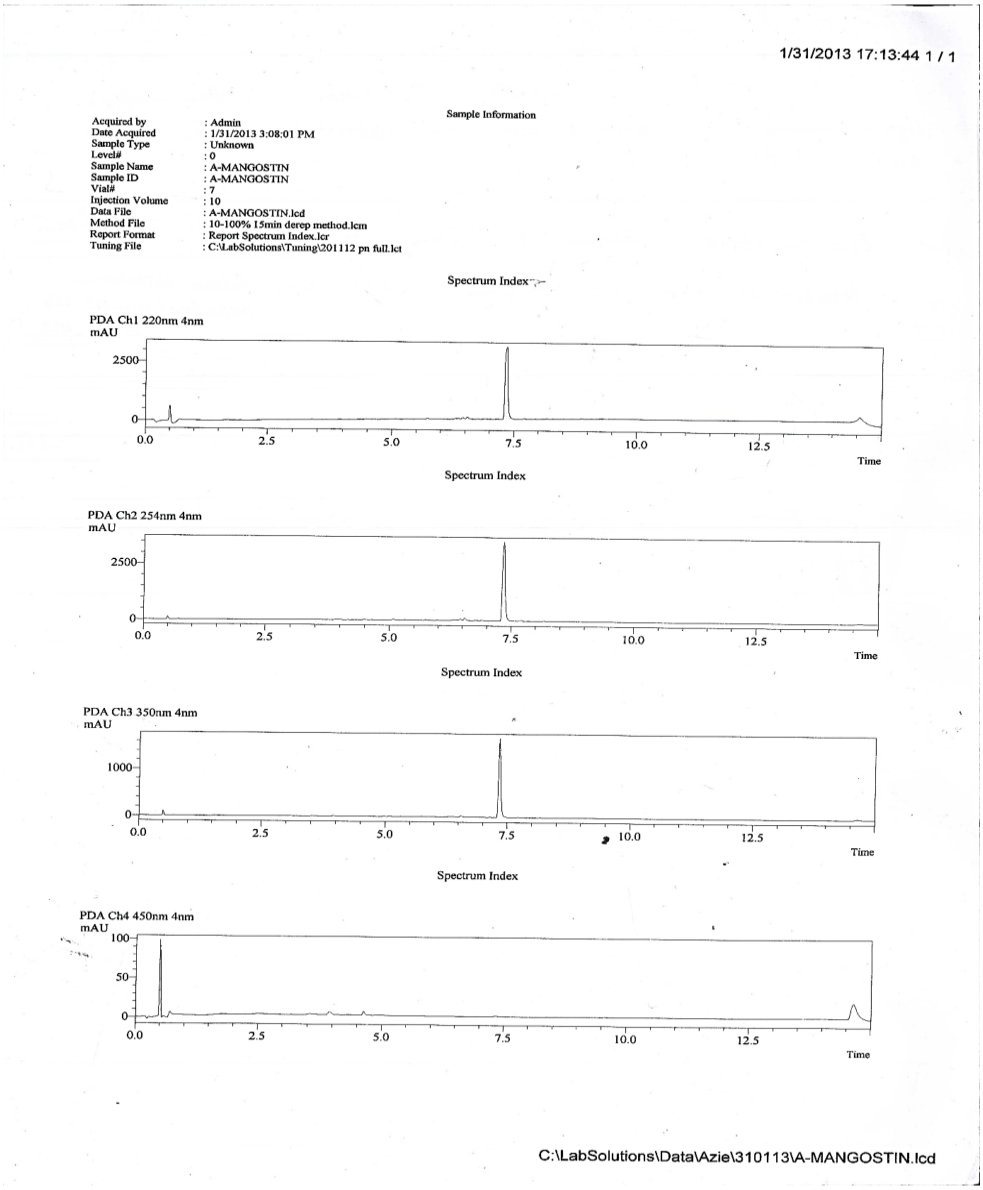

Figure S1. The detection of the peak was at 245 nm wavelength Gilson absorbance detector (UV-VIS165 Gilson USA).
